# Supplementary material for: Multiphasic strain differentiation of atypical mycobacteria from elephant trunk wash
Source: PeerJ. 2015 Nov 10;3:e1367. doi: 10.7717/peerj.1367 (PMC4647574; doi:10.7717/peerj.1367)
Supplement: Table S2 — (A) ANI results for UM_3, UM_11 and M. parascrofulaceum (B) TETRA results for UM_3, UM_11 and M. parascrofulaceum [file peerj-03-1367-s002.doc]

**Supplementary Table 2.** ANI and TETRA results for UM_3, UM_11 and *M. parascrofulaceum*

1. *ANI*

|  | **UM_11** | **UM_3** | **ADNV01** |
| --- | --- | --- | --- |
| **UM_11** | --- | 99.98* | 88.43 |
| **UM_3** | 99.98 | --- | 88.43 |
| **ADNV01** | 88.46 | 88.47 | --- |

ADNVOI, *M. paracrofulaceum*

*Numbers indicate % similarity in 4799 shared genes

1. TETRA

|  | **UM_11** | **UM_3** | **ADNV01** |
| --- | --- | --- | --- |
| **UM_11** | --- | 0.99999 | 0.99678 |
| **UM_3** | 0.99999 | --- | 0.99666 |
| **ADNV01** | 0.99678 | 0.99666 | --- |

ADNVOI, *M. paracrofulaceum*

*Numbers indicate correlation coefficient values
