# Supplementary material for: Multiphasic strain differentiation of atypical mycobacteria from elephant trunk wash
Source: PeerJ. 2015 Nov 10;3:e1367. doi: 10.7717/peerj.1367 (PMC4647574; doi:10.7717/peerj.1367)
Supplement: Table S3 [file peerj-03-1367-s003.doc]

**Supplementary Table 3.** Strain-specific genes in UM_3 and UM_11

| **UM_3 (n=29)** | **UM_11 (n=16)** |
| --- | --- |
| - Penicillin-binding protein 1A/1B - putative HTH-type transcriptional regulator/MT0088 - Release factor glutamine methyltransferase - Histidine--tRNA ligase - Macrocin-O-methyltransferase (TylF) - Elongation factor G - Antiseptic resistance protein - Anti-anti-sigma factor - Transcript cleavage factor greA - Cytochrome c biogenesis factor - Hypothetical protein - Hypothetical protein - Hypothetical protein - Hypothetical protein - Hypothetical protein - Hypothetical protein - Hypothetical protein - Hypothetical protein - Hypothetical protein - Hypothetical protein - Hypothetical protein - Hypothetical protein - Hypothetical protein - Hypothetical protein - Hypothetical protein - Hypothetical protein - Hypothetical protein - Hypothetical protein - Hypothetical protein | - Putative enoyl-CoA hydratase echA8 - Putative membrane protein - Poly(3-hydroxybutyrate) depolymerase - Elongation factor G - Mce family protein - Mce family protein - Cell division protein FtsZ - Acetyl esterase - UDP-galactofuranosyl transferase GlfT1 - GDP-mannose-dependent alpha-(1-6)-phosphatidylinositol dimannoside mannosyltransferase - Hypothetical protein - Hypothetical protein - Hypothetical protein - Hypothetical protein - Hypothetical protein - Hypothetical protein |
