# Supplementary figures and images for: Multiphasic strain differentiation of atypical mycobacteria from elephant trunk wash
Source: PeerJ. 2015 Nov 10;3:e1367. doi: 10.7717/peerj.1367 (PMC4647574; doi:10.7717/peerj.1367)

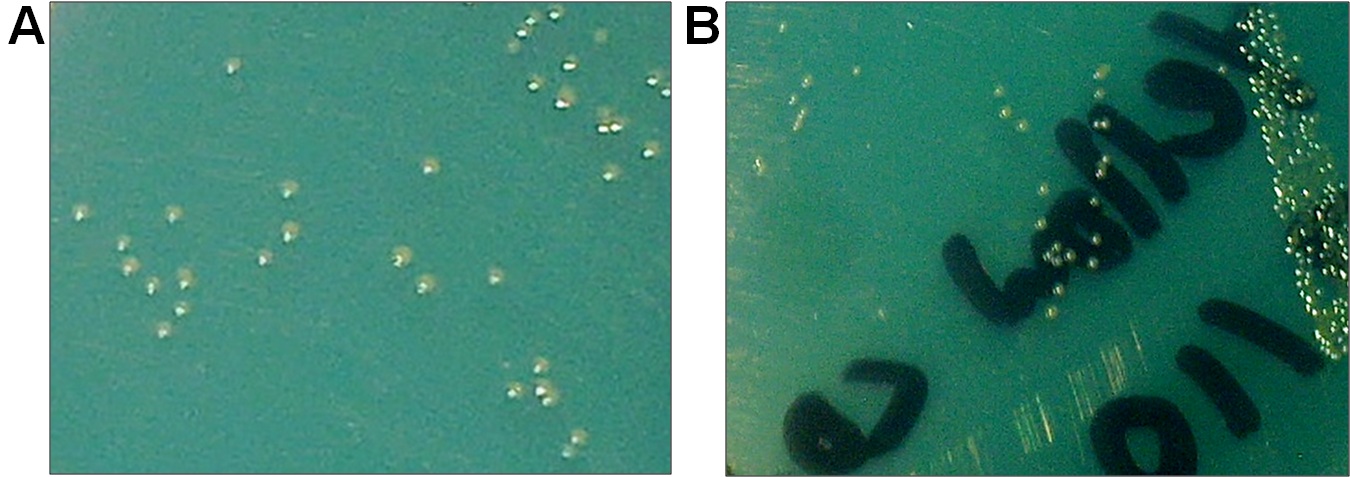

Supplement: Figure S1 [file peerj-03-1367-s004.jpg]

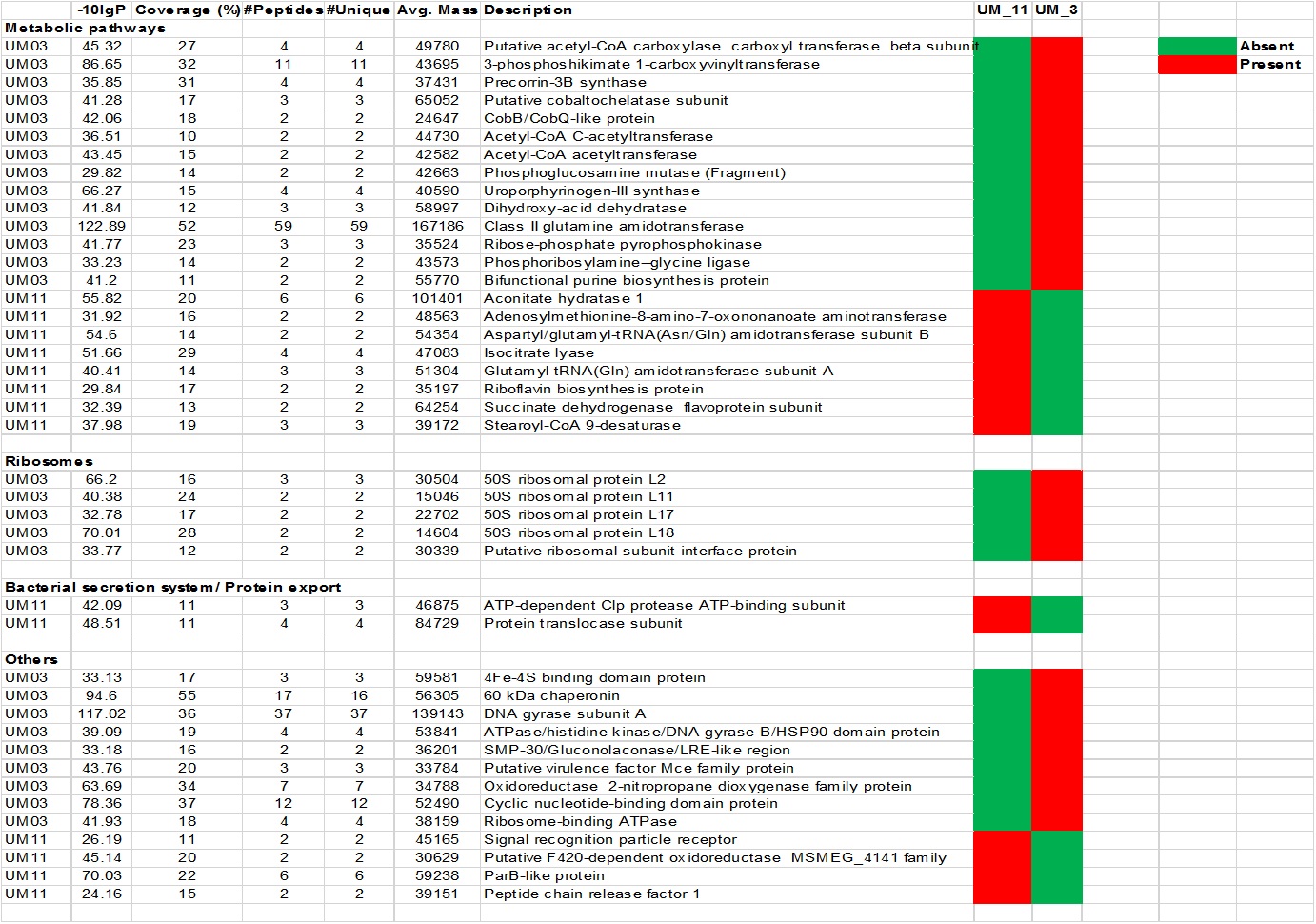

Supplement: Figure S2 [file peerj-03-1367-s005.jpg]

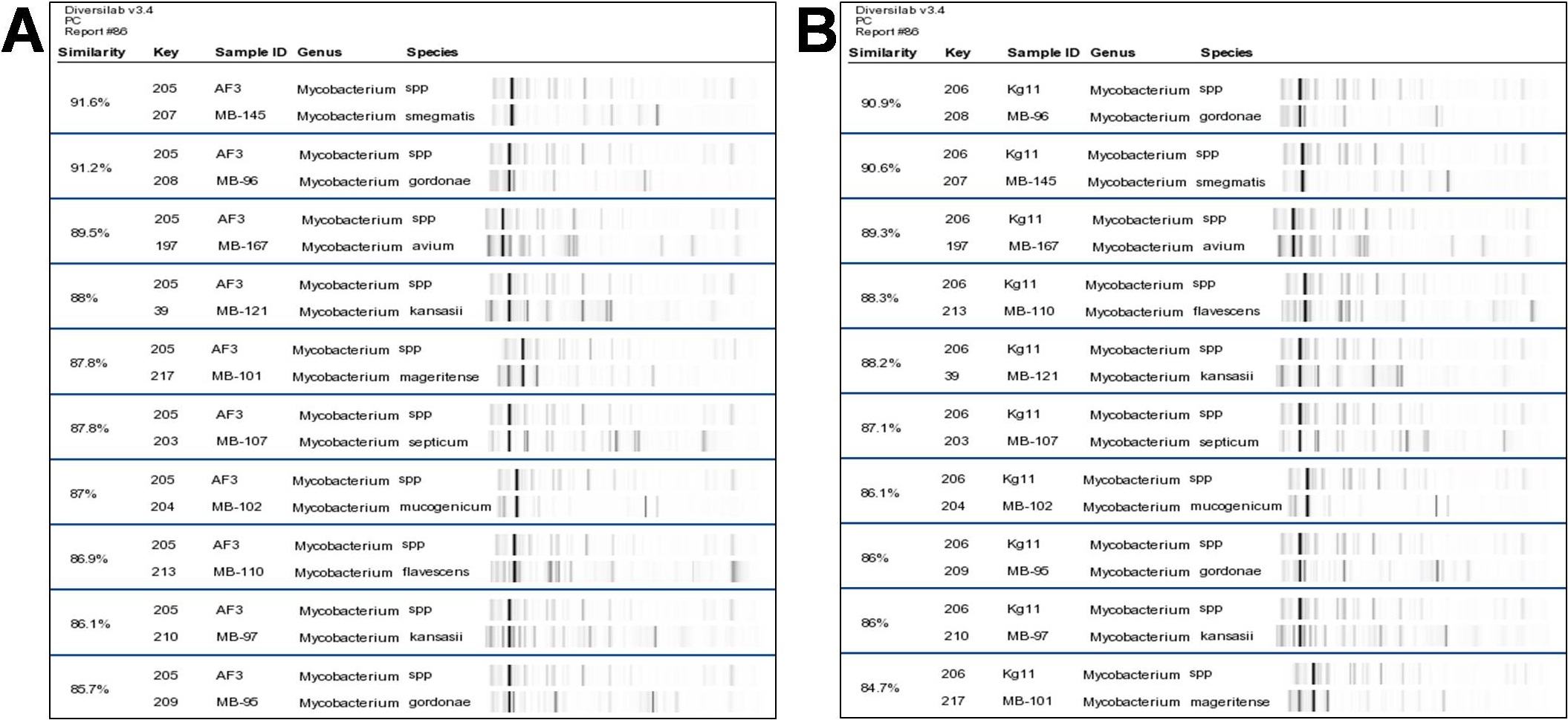

Supplement: Figure S3 [file peerj-03-1367-s006.jpg]
